# Supplementary material for: Birth mode is associated with earliest strain-conferred gut microbiome functions and immunostimulatory potential
Source: Nat Commun. 2018 Nov 30;9:5091. doi: 10.1038/s41467-018-07631-x (PMC6269548; doi:10.1038/s41467-018-07631-x)
Supplement: Supplementary file 1 — Supplementary Information [file 41467_2018_7631_MOESM1_ESM.pdf]

Supplementary Information

Birth mode is associated with earliest strain-conferred gut microbiome functions and immunostimulatory potential

Linda Wampach[1,2,10], Anna Heintz-Buschart[1,3,4,10], Joëlle V. Fritz[1,5,10], Javier Ramiro-Garcia[1], Janine Habier[1], Malte Herold[1], Shaman Narayanasamy[1,6], Anne Kaysen[1,5], Angela H. Hogan[7], Lutz Bindl[5], Jean Bottu[5], Rashi Halder[1], Conny Sjöqvist[8,9], Patrick May[1], Anders F. Andersson[8], Carine de Beaufort[5] and Paul Wilmes[1*]

1. Luxembourg Centre for Systems Biomedicine, University of Luxembourg, avenue des Hauts-Fourneaux 7, 4362 Esch-sur-Alzette, Luxembourg
2. Current address: Laboratoire National de Santé, rue Louis Rech 1, 3555, Dudelange, Luxembourg
3. German Centre for Integrative Biodiversity Research (iDiv) Halle-Jena-Leipzig, Deutscher Platz 5e, 04103 Leipzig, Germany
4. Helmholtz Centre for Environmental Research GmbH – UFZ, Theodor-Lieser-Str. 4, 06120 Halle (Saale), Germany
5. Centre Hospitalier de Luxembourg, rue Nicolas Ernest Barblé 4, 1210 Luxembourg, Luxembourg
6. Megeno S.A., avenue des Hauts-Fourneaux 9, 4362 Esch-sur-Alzette, Luxembourg
7. Integrated BioBank of Luxembourg, rue Louis Rech 1, 3555 Dudelange, Luxembourg
8. KTH Royal Institute of Technology, Science for Life Laboratory, School of Biotechnology, Division of Gene Technology, Tomtebodavägen 23a, 17165 Solna, Sweden
9. Environmental and Marine Biology, Åbo Akademi University, Tykistökatu 6, 20520 Turku, Finland

10. These authors contributed equally to this work.

* Corresponding author: paul.wilmes@uni.lu

The supplementary information file contains supplementary notes, supplementary figures and supplementary references.

Supplementary Notes

Supplementary Note 1	Removal of artefactual sequences from study samples
Supplementary Note 2	General microbiome characteristics of mother-neonate pairs during the first five days of life
Supplementary Note 3	Statistical analyses of metagenomic and 16S rRNA gene amplicon sequencing data
Supplementary Note 4	Multivariate analysis of metagenomic and 16S rRNA gene amplicon sequencing data
Supplementary Note 5	Assessment of LPS purity based on gel staining and reporter cell line assays
Supplementary Note 6	Varying immunostimulatory potential of faecal LPS
Supplementary Note 7	Higher levels of TNF-α and IL-18 in VD neonatal plasma collected 3 days postpartum

Supplementary Figures

Supplementary Figure 1	Removal of artefactual sequences from study samples.
Supplementary Figure 2	Microbial community structures according to sequencing data types.
Supplementary Figure 3	Correlation between sequencing data types and taxonomic composition over the first 5 days postpartum.
Supplementary Figure 4	Assessment of functional potential across sample types and between maternal and neonatal samples.
Supplementary Figure 5	Underrepresentation of the lipopolysaccharide biosynthesis pathway in caesarean-section-delivered neonates being born small for gestational age or not.
Supplementary Figure 6	Underrepresentation of the cationic antimicrobial peptide resistance pathway in caesarean-section-delivered neonates being born small for gestational age or not.
Supplementary Figure 7	Underrepresentation of the ABC transporters pathway in caesarean-section-delivered neonates being born small for gestational age or not.

Supplementary Figure 8	Underrepresentation of the two-component system pathway in caesarean-section-delivered neonates being born small for gestational age or not.
Supplementary Figure 9	Underrepresentation of the bacterial chemotaxis pathway in caesarean-section-delivered neonates being born small for gestational age or not.
Supplementary Figure 10	Underrepresentation of the flagellar assembly pathway in caesarean-section-delivered neonates being born small for gestational age or not.
Supplementary Figure 11	Genetic variation between microbial populations over time.
Supplementary Figure 12	Purity assessment of isolated LPS fractions from neonatal stool.
Supplementary Figure 13	Immune stimulation assays of reporter cell lines after stimulation with isolated LPS from neonatal stool.
Supplementary Figure 14	Cytokine profiles in monocyte-derived dendritic cells after stimulation with isolated LPS from neonatal stool and in neonatal plasma.

Supplementary Notes

Supplementary Note 1 | Removal of artefactual sequences from study samples

The proper curation of artefactual reads from sequencing data of study samples is essential when working with low-biomass microbiome samples such as neonatal faecal samples. For the 16S rRNA gene amplicon sequencing data, any possible effect from putative artefactual reads was limited by applying the methodology previously described in Wampach et al. and thereby focusing on the dominant phylotypes[1]. For metagenomic data, our *in silico* workflow for the identification and removal of artefacts (Fig. 1b) first visualizes the clustering of contigs[2] from the artefact control samples and the study samples (Supplementary Fig. 1a), and subsequently removes contigs from study samples that overlap artefactual contigs, i.e. that fall into the same bin. After subsequent filtering steps and the successful removal of artefactual contigs from all study samples, we observed differences in the number of removed reads according to sample type (Supplementary Fig. 1b). While the impact of read removal by trimming and quality-filtering processes was limited (in average 0.4% of reads were removed across all study samples), the removal of human and artefactual reads had a greater impact on study samples and depended strongly on the sample type. In contrast to maternal stool samples (in average 9% of reads were removed), vaginal swabs contain primarily human mucosal material. Therefore, a large fraction of the extracted DNA from these samples is of human origin and the corresponding sequencing reads were therefore removed from the study samples (in average 98% of reads were removed). This also applied for meconium samples (neonatal faeces collected in the first 24 h), which mainly mirror the perinatal maternal environment[3]. These samples typically present different ratios of human and bacterial DNA contents, compared to later samples, which explains why certain meconium samples were subject to a drastic removal of human reads (in average 69% of reads were removed). In neonatal faecal samples collected on days 3 and 5, removal of human reads decreased subsequently (in average 20% and 13% of reads removed for samples collected on day 3 and day 5 respectively).

Because of the low level of DNA that can be extracted from such samples, meconium samples are prone to over-representation of artefactual DNA introduced during the extraction procedure or preparation of sequence libraries[4,5]. While some meconium samples showed a significant removal of artefactual reads (up to 95.2% of reads were removed), the impact of artefactual removal on later study samples was rather

individual-specific and less dependent on the collection timepoint (Supplementary Fig. 1b; Supplementary Data 2). Generally, artefactual DNA seems to have less impact on neonatal samples at later timepoints when compared to meconium samples, as the percentage of remaining filtered and non-artefactual reads overall increased considerably (from an average of 14% for day 1 to an average of 70% and 64% at days 3 and 5 respectively). This observation further highlights the fact that microbial colonization quickly increases the bacterial load inside the neonatal gut during the first few days postpartum[1].

For the artefact control sample, the combined relative abundance of *Achromobacter xylosoxidans* (39%), *Burkholderia cenocepacia* (30%) and *Burkholderia ambifaria* (13%) was 82%, while the 17 remaining detected taxa each only made up 0.8–1.8% (Supplementary Data 3). The genus *Burkholderia* is a commonly known taxon that contaminates sequencing reagents[5], and contamination by *A. xylosoxidans* has recently been suggested to be derived from contaminated water or scientific reagents[6]. After the removal of the putative artefactual contigs, the maternal and neonatal microbiome samples were largely composed of bona fide reads (maternal faecal samples: 87% of the relative abundance of all detected taxa without any of the putative artefactual taxa; maternal vaginal swab: 98%; neonatal faecal samples from day 1: 89%, day 3: 90% and day 5: 94%; Supplementary Data 3). Notably, metagenomic operational taxonomic units (mOTUs) that were represented in both the artefact control sample and in study samples were significantly overrepresented in the study samples compared with the artefact control sample, suggesting that an actual artefactual origin of these taxa in study samples is highly unlikely. Additionally, the three dominant taxa in the artefact control sample (*Achromobacter xylosoxidans*, *Burkholderia cenocepacia* and *Burkholderia ambifaria*) were largely underrepresented in study samples and only accounted for minor relative abundances within the various study samples (maternal faecal samples: 0.008%; maternal vaginal swab: 1.4%; neonatal faecal samples from day 1: 2.6%, day 3: 0.005% and day 5: 0.01%).

Supplementary Note 2 | General microbiome characteristics of mother-neonate pairs during the first five days of life

When comparing the mean relative abundances for the different genera obtained from both the 16S rRNA gene amplicon and metagenomic sequencing data, and relating to the neonatal gut microbiome during the first 5 days after birth, we generally observed analogous microbial profile compositions (Supplementary Fig. 3a). Also in relation to general microbial colonization and succession patterns, we observed similar results with respect to the different sequencing approaches (Supplementary Fig. 2a and b). While the main trends in terms of diversity (Shannon's diversity index), evenness (Pielou's evenness index) and richness (number of OTUs/mOTUs) were highly comparable between metagenomic and amplicon sequencing data, more pronounced differences were observed for neonatal meconium samples. Faecal samples collected at day 1 postpartum generally showed higher trends of diversity, evenness and most notably richness using 16S rRNA gene amplicon sequencing compared to those inferred from the metagenomic sequencing data. For both types of sequencing, the maternal gut microbiomes generally exhibited higher diversity, evenness and richness compared to those of neonates, and the vaginal swabs were comparatively low for all three indices. Following an initially high diversity and evenness in neonates at day 1, we observed a subsequent drop by day 3, which further stabilized at day 5. The Spearman correlations (calculated between samples collected at days 1-5 from neonates and their respective maternal faecal or vaginal sample) showed an increased similarity of the taxonomic profiles of vaginally-delivered neonates (VD) to those of the maternal gut microbiomes compared to caesarean-section-delivered (CSD) neonates (Supplementary Fig. 2c and d). However, no differences according to delivery mode were identified studying the correlations in relation to the sequencing data obtained from the maternal vaginal samples.

Supplementary Note 3 | Statistical analyses of metagenomic and 16S rRNA gene amplicon sequencing data

To identify differences between the birth modes (and SGA status) and the different collection time points postpartum, we performed Wilcoxon rank-sum tests (without multiple-alignment adjustments) on the sum-normalized relative abundances of metagenomic and 16S rRNA gene amplicon sequencing data (Supplementary Data 7 and 8). Although several statistically significant differences were found, we only considered genera and OTUs that showed the same trends for both CSD and CSD+SGA neonates and had a P-value below 0.05. In VD neonates, compared to both CSD and CSD+SGA, the metagenomic data resolved a higher relative abundance of the species *Bacteroides dorei/vulgatus* (day 5 VD vs CSD, $P = 2.5 \times 10^{-2}$ and VD vs CSD+SGA, $P = 1.2 \times 10^{-2}$), as well as the specific *Bacteroides dorei/vulgatus* mOTU (day 5 VD vs CSD, $P = 1.4 \times 10^{-2}$ and VD vs CSD+SGA, $P = 6.7 \times 10^{-3}$). Based on the 16S rRNA gene amplicon sequencing data, significantly higher relative abundances of the genus *Bacteroides* were observed in VD neonates at days 3 and 5 (day 3, $P = 7.8 \times 10^{-3}$ and $P = 1.2 \times 10^{-2}$; day 5, $P = 1.2 \times 10^{-3}$ and $P = 5.1 \times 10^{-3}$, respectively). The genus *Parabacteroides* was significantly increased ($P = 4.2 \times 10^{-3}$ and $P = 2.8 \times 10^{-2}$), while *Rothia* was found to be significantly decreased in VD neonates ($P = 1.8 \times 10^{-2}$ and $P = 2.2 \times 10^{-4}$). A total of 10 OTUs assigned to the genus *Staphylococcus* were significantly increased in CSD±SGA neonates, while OTU 243 assigned to *Bacteroides* was significantly increased in VD neonates at days 3 and 5 (day 3, $P = 2.3 \times 10^{-2}$ and $P = 2.5 \times 10^{-2}$; day 5, $P = 2.5 \times 10^{-3}$ and $P = 9.4 \times 10^{-3}$, respectively).

Supplementary Note 4 | Multivariate analysis of metagenomic and 16S rRNA gene amplicon sequencing data

The administration of antibiotics to mothers prior to caesarean section delivery is often (e.g. in Luxembourg) mandatory, resulting in delivery by caesarean section and the maternal intake of antibiotics to be commonly coinciding factors. Additionally, vaginally delivering mothers that are positively screened for group B *Streptococcus* infection receive antibiotics prior to delivery to reduce the risk of infection in neonates. For these reasons, most mothers were administered antibiotics in this study, for both delivery modes. As other factors (e.g. day after delivery, gestational age, feeding regime, etc.) have been suggested to impact neonatal gut microbiome colonization as well, we used a multivariate analysis (MaAsLin[7]) to identify differentially abundant taxa for both metagenomic and 16S rRNA gene amplicon sequencing data (Supplementary Data 9). After correcting for the respective effects of all the above-mentioned variables for the metagenomic sequencing data, three mOTUs were associated with delivery mode (CSD±SGA) and at the same time maternal antibiotics intake, namely *Bacteroides fragilis* (for all conditions: $Q = 8.1 \times 10^{-3}$), *Bacteroides xylanisolvens* (for all conditions: $Q = 8.1\times 10^{-3}$) and *Parabacteroides merda* (for all conditions: $Q = 8.1 \times 10^{-3}$). In contrast, when correcting the different factors for the larger cohort screened by 16S rRNA gene amplicon sequencing using MaAsLin[7], delivery mode had a distinct driving effect on the earliest microbiome. More specifically, *Bacteroides* was significantly decreased in CSD±SGA neonates ($Q = 2.6 \times 10^{-3}$ and $Q = 2.9 \times 10^{-2}$) as well as Bacteroidaceae (CSD; $Q = 3.9 \times 10^{-2}$). One OTU of *Escherichia-Shigella* was significantly decreased in CSD±SGA neonates (both $Q = 3.6 \times 10^{-2}$). Two OTUs belonging to *Bacteroides* ($Q = 3.6 \times 10^{-2}$ and $Q = 3.9 \times 10^{-2}$) and two OTUs belonging to *Bifidobacterium* ($Q = 1.6 \times 10^{-2}$ and $Q = 4.8 \times 10^{-2}$) were significantly decreased in CSD neonates while one OTU belonging to *Staphylococcus* was significantly increased in CSD neonates relative to VD ($Q = 2.3 \times 10^{-2}$). Feeding regime was found to be a potential contributing factor of the relative abundances of *Trichococcus* (formula feeding and mixed feeding regime; $Q = 3.6 \times 10^{-3}$ and $Q = 4.6 \times 10^{-2}$), *Escherichia-Shigella* (mixed feeding regime; $Q = 1.3 \times 10^{-2}$) and one OTU belonging to *Rothia* (formula feeding regime; $Q = 2.9 \times 10^{-4}$). The genus *Proteus* ($Q = 1.0 \times 10^{-2}$) was associated with maternal antibiotics intake and multiple genera and OTUs were associated with faecal samples collected at day 1 postpartum. Although we cannot exclude an effect of maternal antibiotic exposure or minor effects of feeding regime or collection time point on the taxonomic composition of the

neonatal gut microbiome, the main differences in microbial taxa in both datasets (16S rRNA gene amplicon and metagenomic sequencing data) were clearly due to delivery mode.

An additional analysis to test for differentially abundant taxa associated with delivery mode was performed using ANCOM[8]. The results further confirmed the significantly decreased relative abundances of *Bacteroides*, *Escherichia-Shigella, Bifidobacterium* and *Parabacteroides* in CSD±SGA neonates, while *Staphylococcus* was significantly increased in CSD±SGA neonate (all these results were based on the statistical analyses based on Wilcoxon rank-sum tests, as well as the multivariate analyses). Our results demonstrate that although several minor trends inside the earliest neonatal gut microbiome were associated with distinct neonatal or maternal factors, the observed fundamental changes in earliest community compositions were first and foremost due to the delivery mode.

Supplementary Note 5 | Assessment of LPS purity based on gel staining and reporter cell line assays

To assess the LPS purity, we performed detailed characterizations of the obtained LPS fractions. First, we used agarose gel electrophoresis to exclude the presence of DNA contamination (Supplementary Fig. 12a) with the subsequent extraction of DNA from excised agarose bands and measurement of TNF-α in the supernatant of MoDCs upon stimulation (Supplementary Fig. 12b). Our results confirmed that DNA contamination did not considerably contribute to the observed immune activation of MoDCs. Second, we used polyacrylamide gel electrophoresis combined with Coomassie staining in order to successfully exclude the presence of proteins (Supplementary Fig. 12c). Third, we visualized LPS using polyacrylamide gel electrophoresis followed by silver staining (Supplementary Fig. 12d).

To further assess the immunogenicity as well as the purity of the LPS fractions, we performed additional stimulation assays using specific HEK-Blue™ reporter cell lines which overexpressed either hTLR4, hTLR2, hNOD1 or hNOD2 (Supplementary Fig. 13a to d). In summary, when LPS was used at a defined concentration for all samples, only hTLR4 was activated and not hTLR2, nor hNOD1 or hNOD2; if isolated LPS was used at sample-specific concentrations to mimic realistic *in vivo* conditions, LPS mixtures were still relatively pure and comparable to commercially available LPS (Sigma) as hNOD1 and hNOD2 were in both cases not activated. In samples that were highly enriched in LPS also hTLR2 was activated, which was also the case for the commercially available, pure LPS. Finally, in case a minimal amount of endotoxin unit (EU) of LPS was used to stimulate dendritic cells, the TNF-α response did not correlate with a linearly increasing amount of both LPS and TNF-α, suggesting that indeed the composition of the different isolated LPS mixtures does play the dominant role in the observed immune responses.

Supplementary Note 6 | Varying immunostimulatory potential of faecal LPS

In the framework of LPS isolation, the same amount of material for all neonatal stool samples and the exact same extraction protocol were used. The rationale for the subsequent experiments was that if MoDCs from the same donor were treated with the exact same volume of LPS extract (independent of the concentration of LPS present), the microbial LPS load of a given sample is representative of what immune cells would be exposed to in the *in vivo* situation, thereby reflecting the immunostimulatory potential of a given sample at 3 days postpartum. After monocyte-derived dendritic cells (MoDCs) from twelve adult donors were incubated with LPS isolated from neonatal faecal samples, TNF-α was found at elevated levels when cells were exposed to LPS from VD faecal samples (Fig. 4a; Supplementary Data 13). The observed levels of TNF-α mostly correlated with the relative abundance of Gram-negative bacteria (Fig. 4a, Supplementary Fig.3b). LPS from VD neonate C117, in whom the microbiome displayed lower presence of Gram-negative bacteria based on 16S rRNA gene amplicon sequencing data and the lowest amounts of *E. coli* according to qPCR measurements, also had the lowest immunostimulatory potential among all VD neonates, and triggered a negligible cytokine response. Although a higher *E. coli* abundance appeared to coincide with an increased immunostimulatory potential of the isolated LPS, the proportion of *E. coli* alone was not sufficient to explain the lack of immunostimulative effects in CSD (±SGA) neonates. More specifically, in five cases of faecal samples collected from CSD±SGA, the absolute abundance of *E. coli* was at least 25-fold increased when compared to the faecal sample collected from VD neonate C007, which triggered an immune response (Fig. 4a). However, the isolated LPS from these five samples did not result in any considerable immune response in terms of TNF-α production. Additionally, in CSD neonate C121, the microbiome was depleted of *E. coli*, while the extracted LPS fraction still triggered an immune response. At the same time, CSD+SGA neonate C119 was depleted of *E. coli* but had a high proportion of Gram negative bacteria overall, while the LPS extract only triggered a minimal immune response in some of the MoDCs from adult donors. Collectively, these observations indicate that the composition of the isolated LPS fractions has an important role in their activity as well.

Additionally, we employed a method for LPS stimulation, whereby the amount of LPS to stimulate MoDCs was normalized to the bacterial load. After monocyte-derived dendritic cells (MoDCs) from four adult donors were incubated with LPS isolated

from neonatal faecal samples, in total seven cytokines (CXCL8/IL-8, IL-1β, IL-6, IL-10, IL-12p70, IL-18 and TNF-α) were found at elevated levels in response to LPS from the gut microbiota of VD neonates compared to CSD (± SGA) neonates, most notably TNF-α and IL-18 (Supplementary Fig. 14, Supplementary Data 15 and 16). Again, evidence was found that the composition of the LPS had an important role in its activity. The faecal samples of the neonates C109 (VD) and C121 (CSD) had approximately the same levels of bacteria (76 ng and 73 ng of bacterial DNA/mg stool, respectively), but the estimated number of endotoxin units was approximately double the amount for the CSD neonate (24 versus 46) and yet its LPS triggered a far less pronounced immune response compared to the one observed for C109. Taken together, both approaches conveyed the same trends, while also reflecting the previously demonstrated importance of the specific composition of LPS for immune stimulation[9].

Supplementary Note 7 | Higher levels of TNF-α and IL-18 in VD neonatal plasma collected 3 days postpartum

After measuring the immune stimulation of monocyte-derived dendritic cells (MoDCs) by LPS and assessing the cytokine levels in neonatal blood plasma collected at day 3 after birth (Supplementary Data 17), two cytokines were observed to be differentially abundant according to birth mode (Fig. 4b). The concentrations of TNF-α generally increase in response to LPS exposure[59], and in our study, TNF-α was in the range as previously measured in healthy infants aged 0–3 months[23]. Furthermore and analogous to earlier findings[22], we observed a higher level of TNF-α in VD neonates (FDR-adjusted Wilcoxon rank-sum test $P = 3.0 \times 10^{-2}$; Fig. 4b). IL-18 is an important cytokine in natural and acquired immunity[60] and has a protective role against infections caused by pathogens[61]. It is induced by microbial products such as LPS and by exposure to TNF-α[62]. The levels of IL-18 were significantly increased in VD neonates (FDR-adjusted Wilcoxon rank-sum test $P = 2.4 \times 10^{-3}$; Fig. 4b). Taken together, the statistically higher levels of TNF-α and IL-18 in the blood of VD neonates as well as the same cytokines being elevated after stimulation of the MoDCs with LPS isolated from the stool of VD neonates (Fig. 4a; Supplementary Fig. 14), indicates that early LPS-driven immune stimulation is more pronounced in VD neonates compared to CSD (± SGA) neonates.

Supplementary Figures

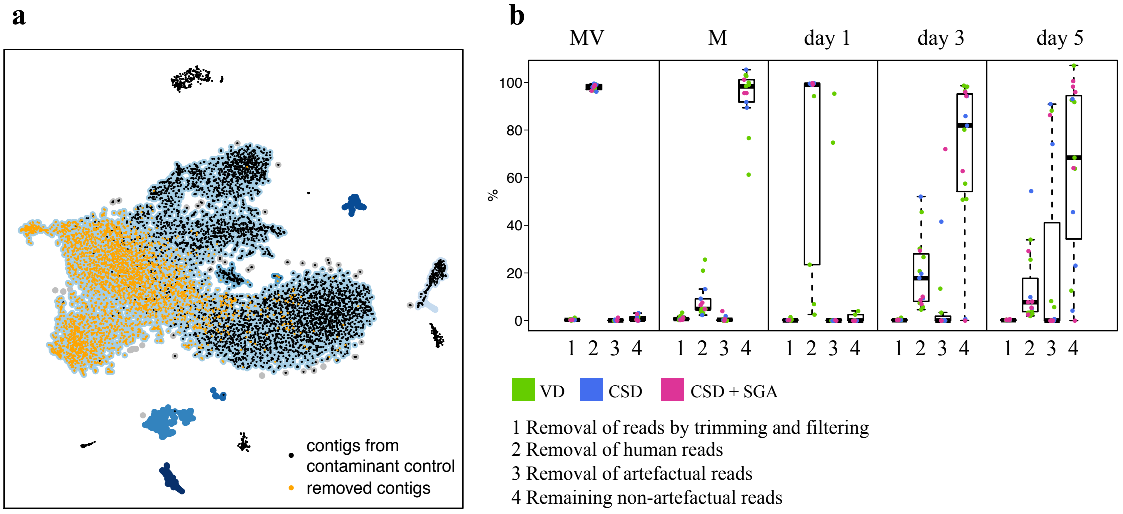

Supplementary Figure 1 | Removal of artefactual sequences from study samples. **a**, Visualization of the result of clustering contigs from contamination controls with contigs of one sample (V3_C106; collected at day 5, VD neonate). Each dot represents one contig. The contigs that were subsequently removed because they overlapped with the clusters formed by sequences from the contaminant control are marked with an orange-coloured core. **b**, Barplot showing the retained proportion of reads removed after different curation steps (1-4) for each sample (separated according to sample origin). The samples are coloured according to birth mode and small for gestational age (SGA) status. VD, vaginal delivery; CSD, caesarean-section delivery; M, maternal faeces, MV, maternal vaginal swab; day 1/day 3/day 5, neonatal faeces. Boxplots: centre line – median, bounds – first and third quartile, whisker <= 1.5 x interquartile range.

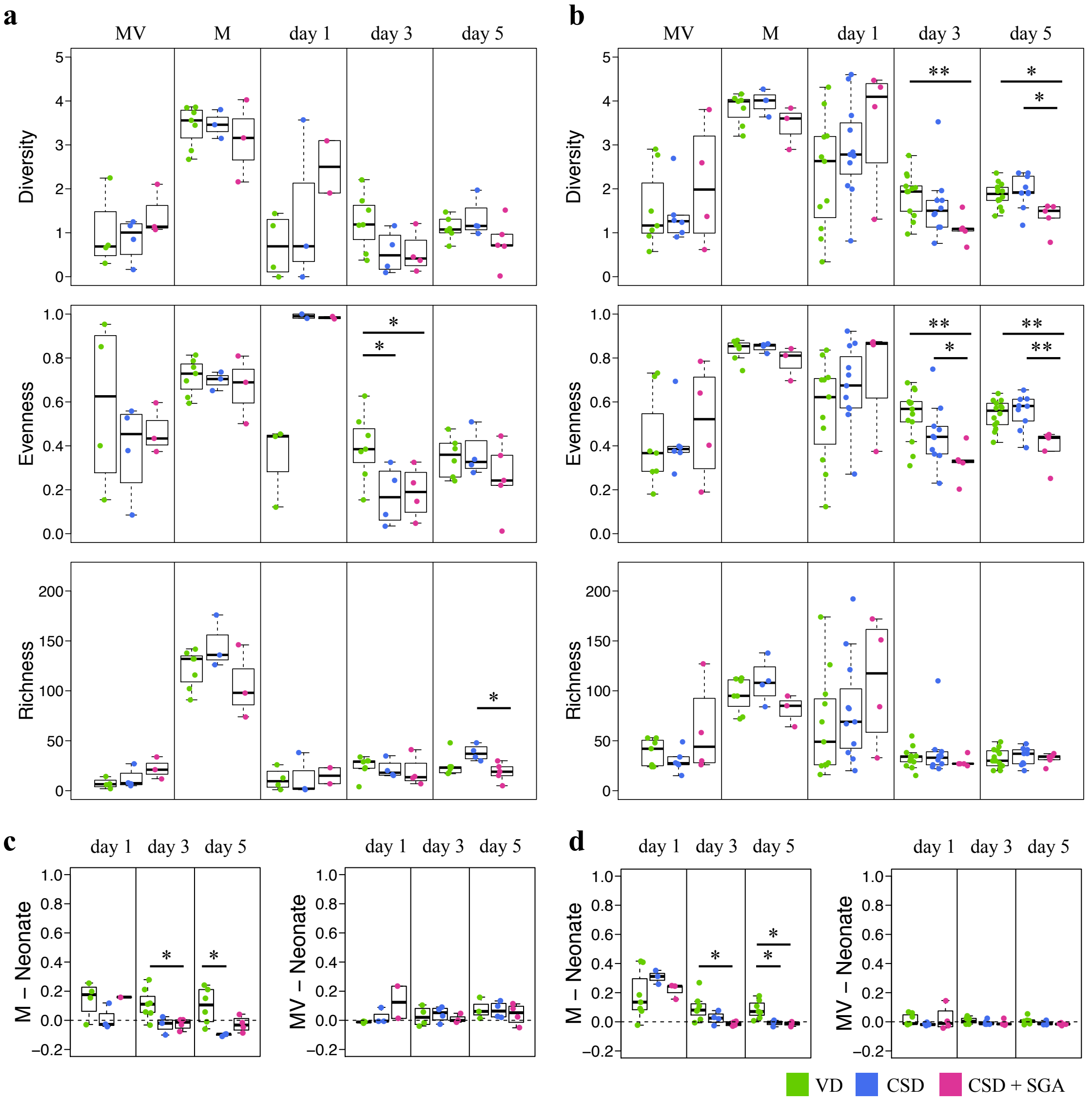

Supplementary Figure 2 | Microbial community structures according to sequencing data types. a–b, Diversity (Shannon's diversity index), evenness (Pielou's evenness index) and richness (number of operational taxonomic units) of gut microbial communities based on (**a**) metagenomic sequencing data and (**b**) 16S rRNA gene amplicon sequencing data . **c–d**, Spearman's correlation coefficients of the taxonomic profiles of neonatal gut microbiota (days 1–5) compared with maternal stool (M) or vaginal (MV) samples, based on (**c**) metagenomic data and (**d**) 16S rRNA gene amplicon sequencing data. The samples are coloured according to birth mode and small for gestational age (SGA) status. Comparison by Wilcoxon rank-sum test with multiple testing adjustment; *false discovery rate (FDR)-adjusted *P* <0.05 and **(FDR)-adjusted *P* <0.01. VD, vaginal delivery; CSD, caesarean-section delivery; M, maternal faeces, MV, maternal vaginal swab; day 1/day 3/day 5, neonatal faeces. Boxplots: centre line – median, bounds – first and third quartile, whisker <= 1.5 x interquartile range.

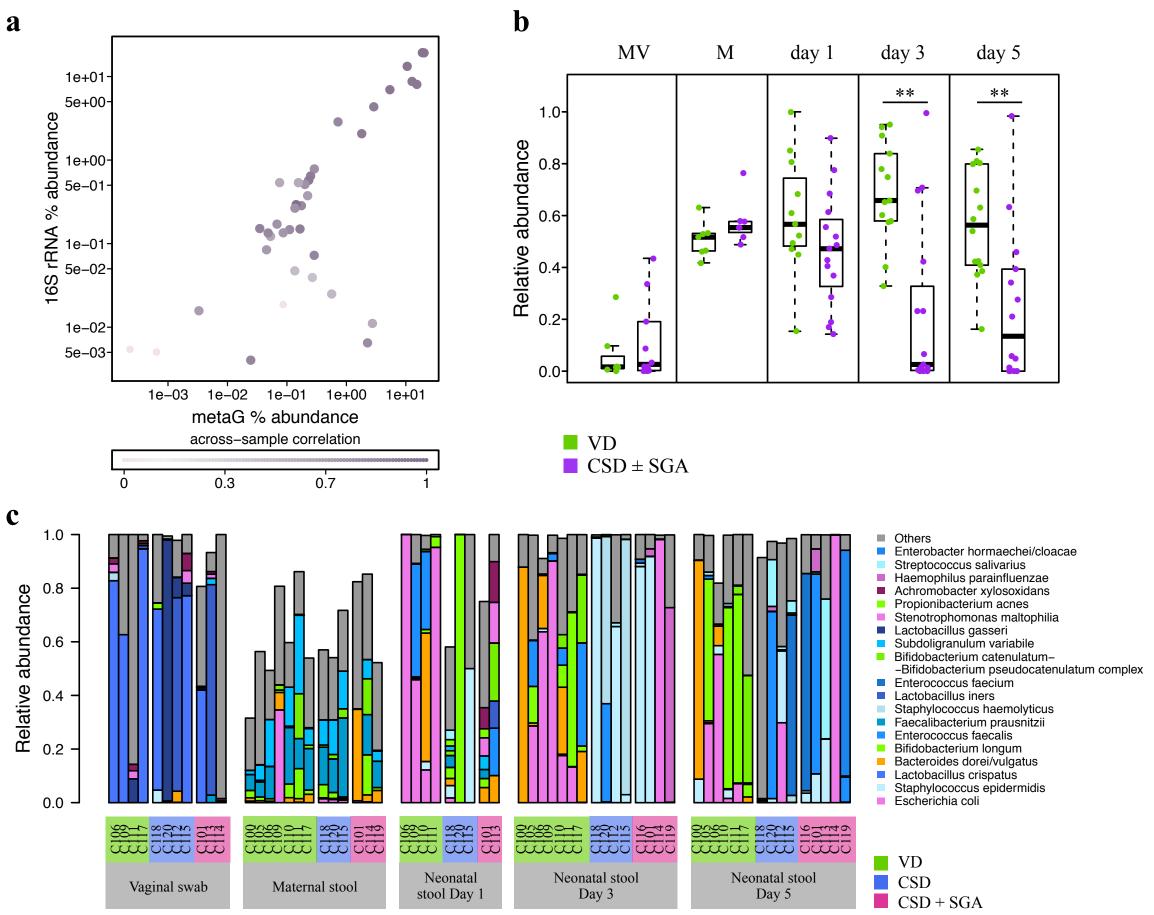

Supplementary Figure 3 | Correlation between sequencing data types and taxonomic composition over the first 5 days postpartum. a, Comparison of mean relative abundance of genera obtained from metagenomic operational taxonomic units (mOTUs) inferred from metagenomic reads[10] and OTUs inferred from 16S rRNA gene amplicon sequencing data of neonatal faecal samples. **b**, The relative abundance of Gram negative bacteria assessed by 16S rRNA gene amplicon sequencing, according to birth mode (VD vs CSD (± SGA)) in maternal samples and over the first 5 days postpartum for the neonatal samples. Boxplots: centre line – median, bounds – first and third quartile, whisker <= 1.5 x interquartile range. Comparison by Wilcoxon rank-sum test with multiple testing adjustment; *false discovery rate (FDR)-adjusted *P* <0.05 and **(FDR)-adjusted *P* <0.01. **c**, Relative abundance of mOTUs. mOTUs of individual species are grouped, and taxa that do not belong to the 19 most abundant species are regrouped as ‘Others’. Only taxa that were assigned at the species level are represented; unknown species levels are regrouped and represented in white. Taxa are colour-coded at the phylum rank. Neonates C115 and C116 are twins. The samples are coloured according to birth mode and small for gestational age (SGA) status. VD, vaginal delivery; CSD, caesarean-section delivery; M, maternal faeces, MV, maternal vaginal swab; day 1/day 3/day 5, neonatal faeces.

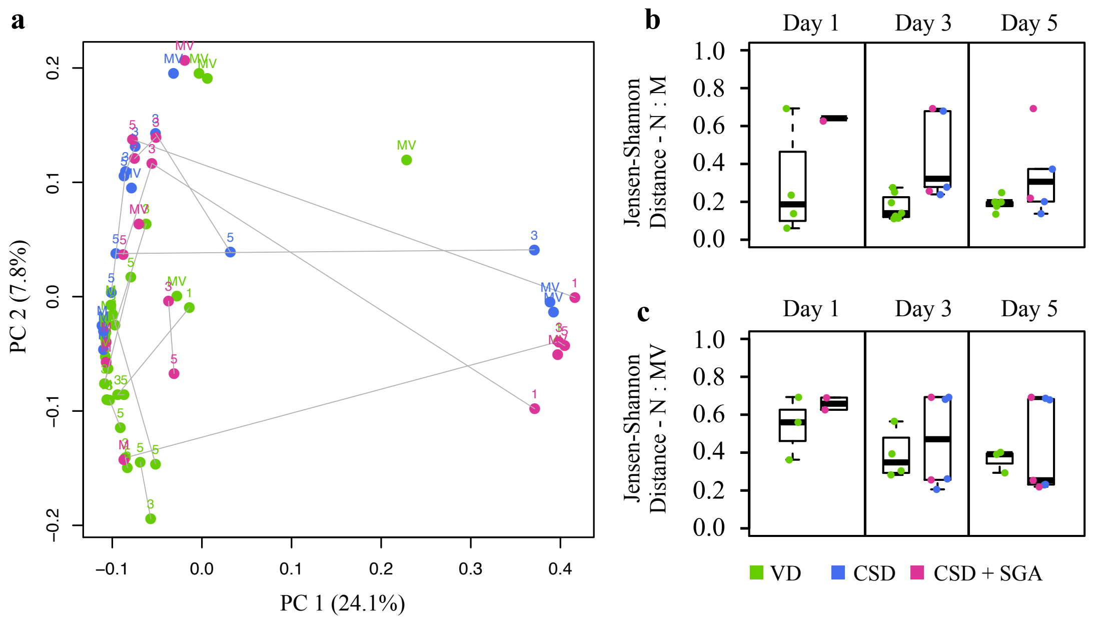

Supplementary Figure 4 | Assessment of functional potential across sample types and between maternal and neonatal samples. a, Principal coordinate analysis of Jensen-Shannon divergence was generated for the functional data of all different sample types. Lines connect samples which originated from the same neonate according to the order of sampling. **b,** Jensen-Shannon divergences of the functional profiles of the neonatal gut microbiomes to those of the respective maternal gut microbiomes. **c,** Jensen-Shannon divergences of the functional profiles of the neonatal gut microbiomes to those of the respective maternal vaginal microbiomes. Samples are coloured according to birth mode and small for gestational age (SGA) status. VD, delivery; CSD, caesarean-section delivery; M, maternal faeces; MV, maternal vaginal swab: N, neonatal faeces collected at day1 / day 3 / day 5. Boxplots: centre line – median, bounds – first and third quartile, whisker <= 1.5 x interquartile range.

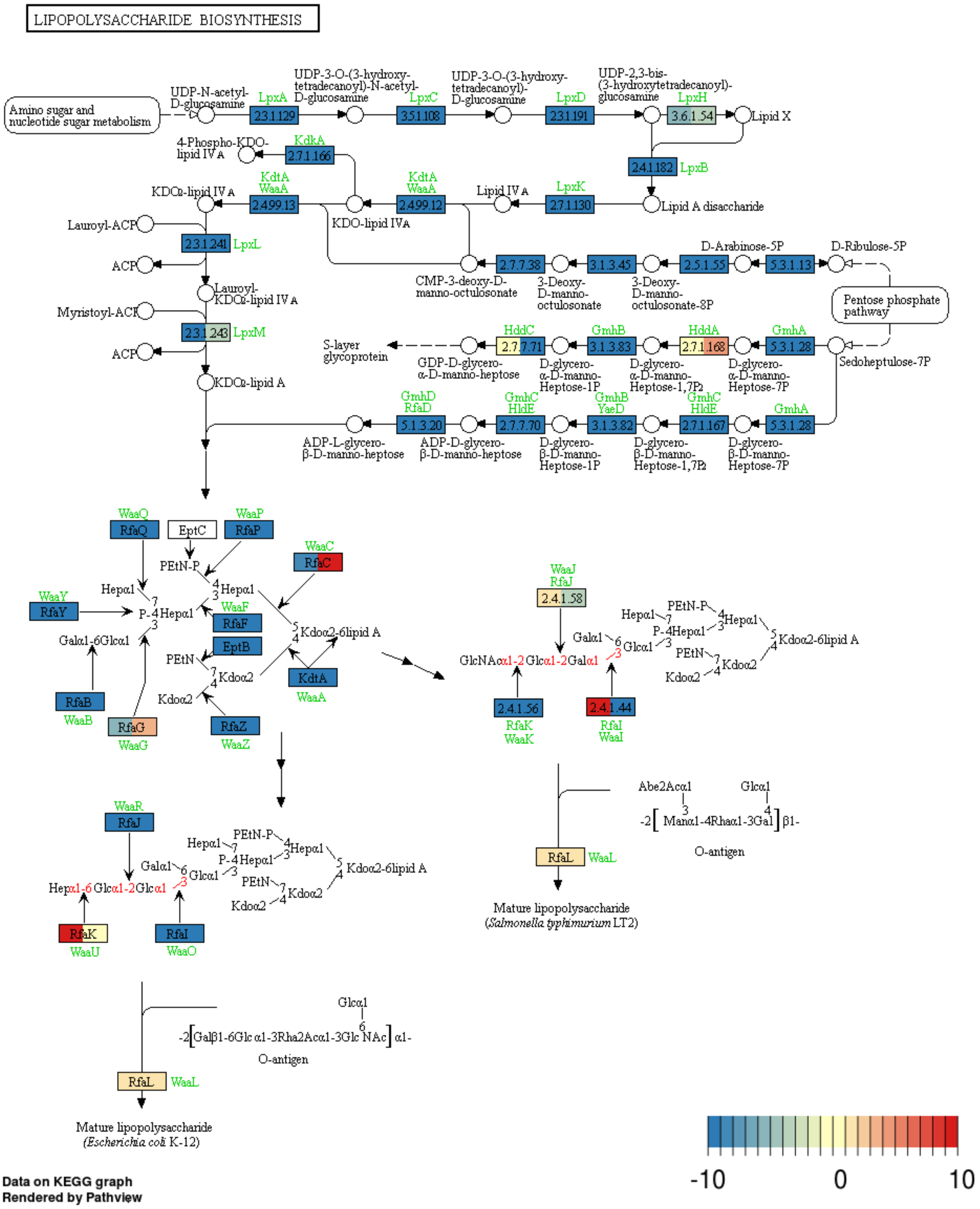

Supplementary Figure 5 | Underrepresentation of the lipopolysaccharide biosynthesis pathway in caesarean-section-delivered neonates being born small for gestational age or not. Enzymes are depicted by boxes coloured according to the $\log_2$ fold changes (see colour key at bottom-right corner of the plot) obtained from the relative abundance of genes in gut metagenomes on days 3 and 5 in caesarean-section-delivered neonates (left part of the box) and additionally born small for gestational age (right part of the box), compared with vaginally delivered neonates. Log_2 fold changes were calculated with the R package ‘DESeq2’[11] and the *pathview* R package[12] was used for plotting.

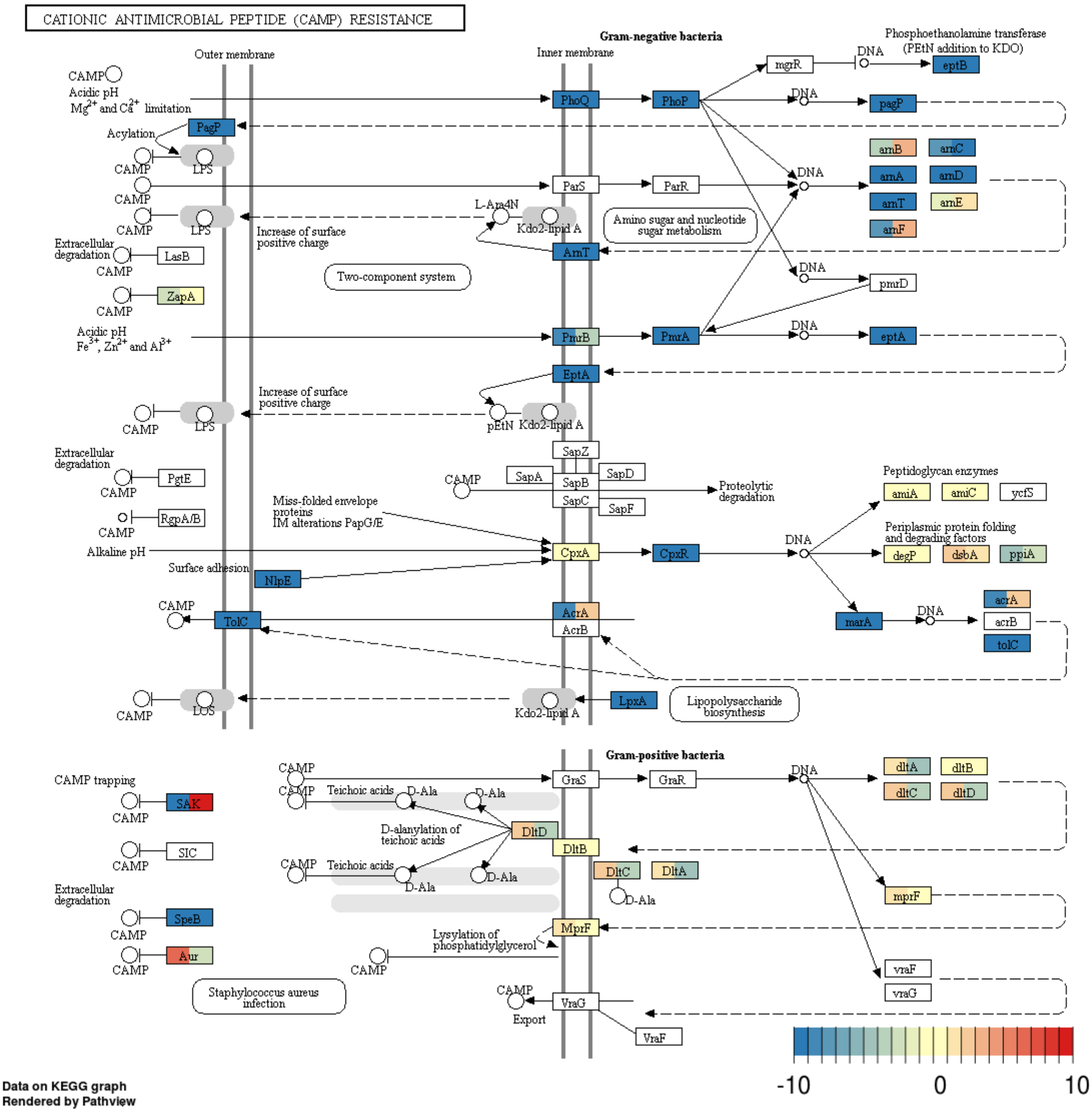

Supplementary Figure 6 | Underrepresentation of the cationic antimicrobial peptide resistance pathway in caesarean-section-delivered neonates being born small for gestational age or not. Enzymes are depicted by boxes coloured according to the $\log_2$ fold changes (see colour key at bottom-right corner of the plot) obtained from the relative abundance of genes in gut metagenomes on days 3 and 5 in caesarean-section-delivered neonates (left part of the box) and additionally born small for gestational age (right part of the box), compared with vaginally delivered neonates. Log_2 fold changes were calculated with the R package 'DESeq2'[11] and the *pathview* R package[12] was used for plotting.

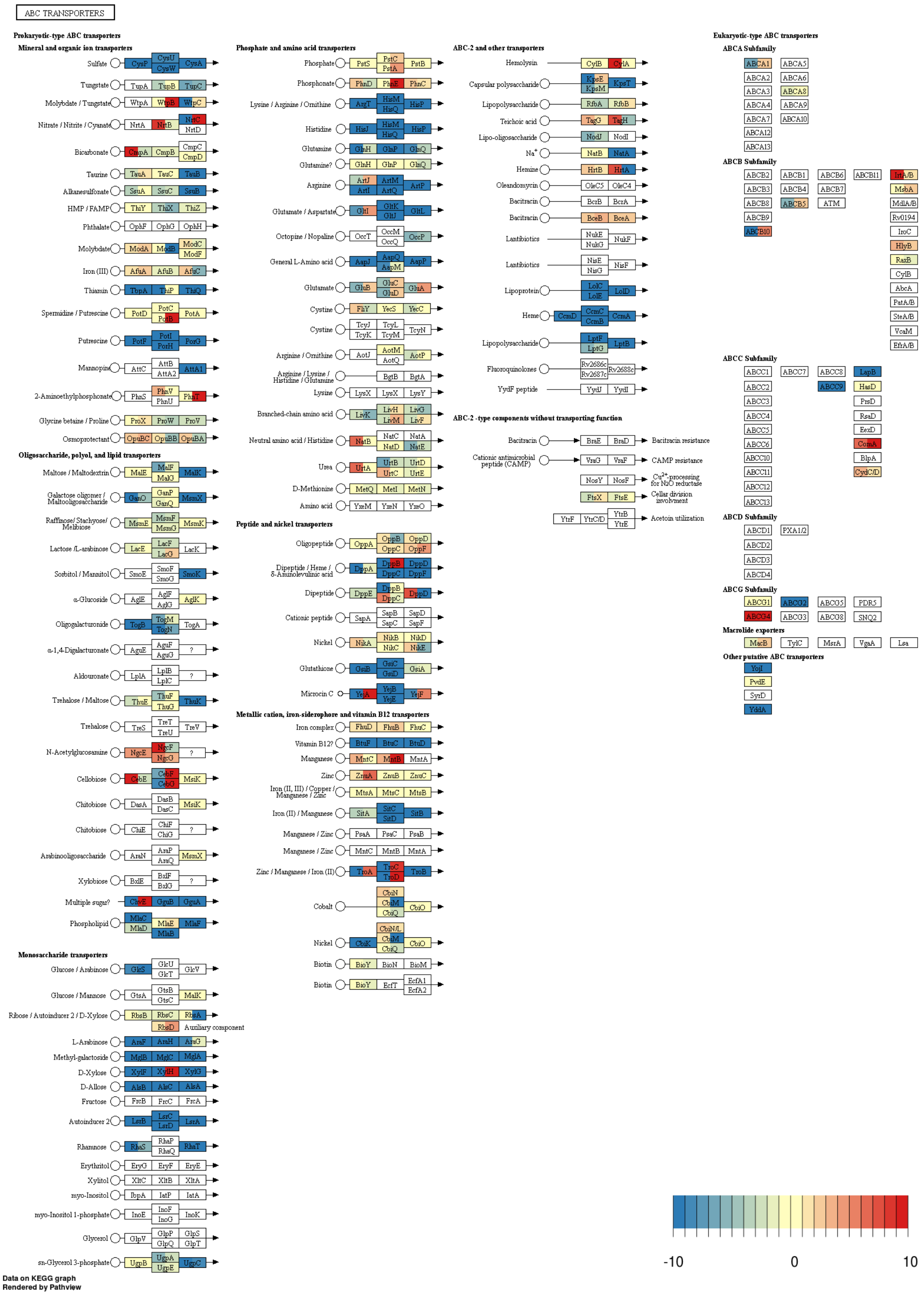

Supplementary Figure 7 | Underrepresentation of the ABC transporters pathway in caesarean-section-delivered neonates being born small for gestational age or not. Enzymes are depicted by boxes coloured according to the $\log_2$ fold changes (see colour key at bottom-right corner of the plot) obtained from the relative abundance of genes in gut metagenomes on days 3 and 5 in caesarean-section-delivered neonates (left part of the box) and additionally born small for gestational age (right part of the box), compared with vaginally delivered neonates. Log_2 fold changes were calculated with the R package ‘DESeq2’[11] and the *pathview* R package[12] was used for plotting.

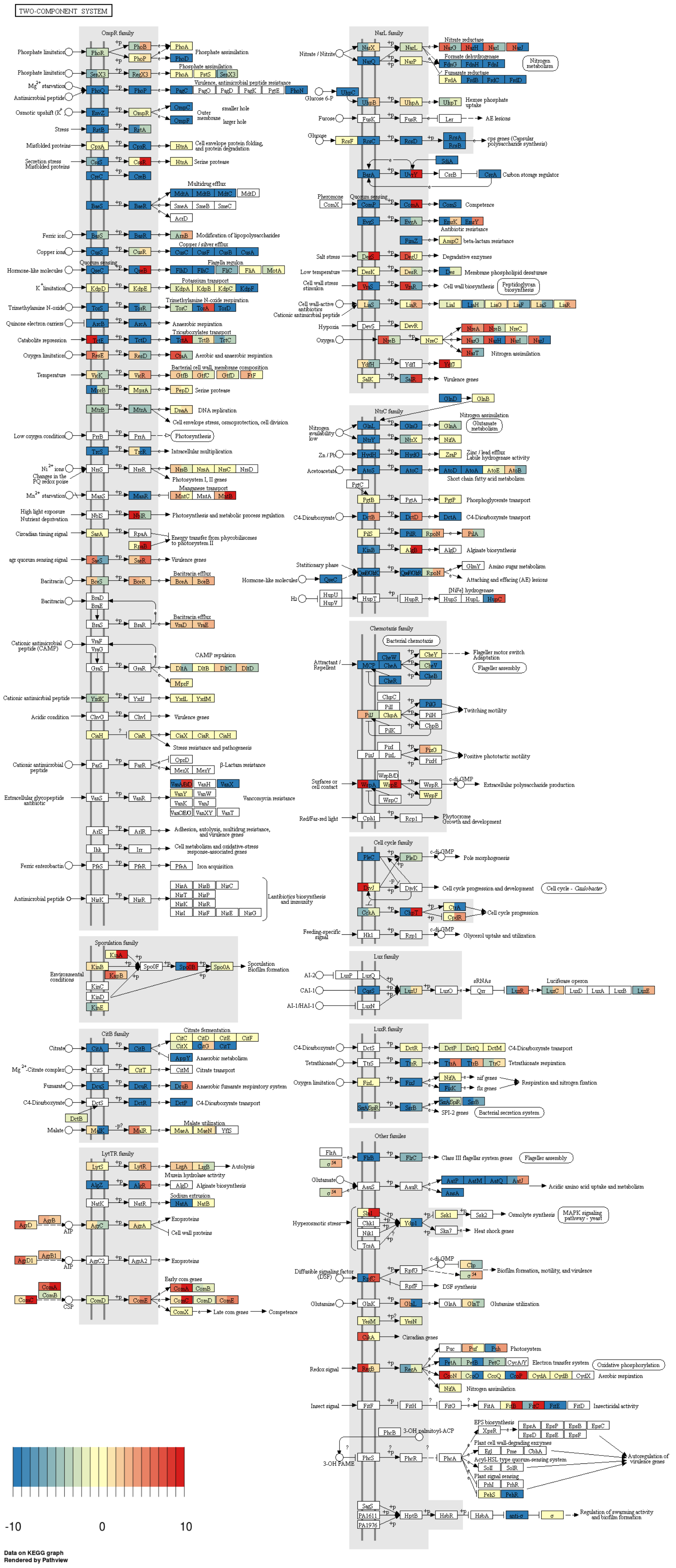
TWO-COMPONENT SYSTEM
-10
0
10
Data on KEGG graph
Rendered by Pathview

Supplementary Figure 8 | Underrepresentation of the two-component system pathway in caesarean-section-delivered neonates being born small for gestational age or not. Enzymes are depicted by boxes coloured according to the $\log_2$ fold changes (see colour key at bottom-left corner of the plot) obtained from the relative abundance of genes in gut metagenomes on days 3 and 5 in caesarean-section-delivered neonates (left part of the box) and additionally born small for gestational age (right part of the box), compared with vaginally delivered neonates. Log_2 fold changes were calculated with the R package 'DESeq2'[11] and the *pathview* R package[12] was used for plotting.

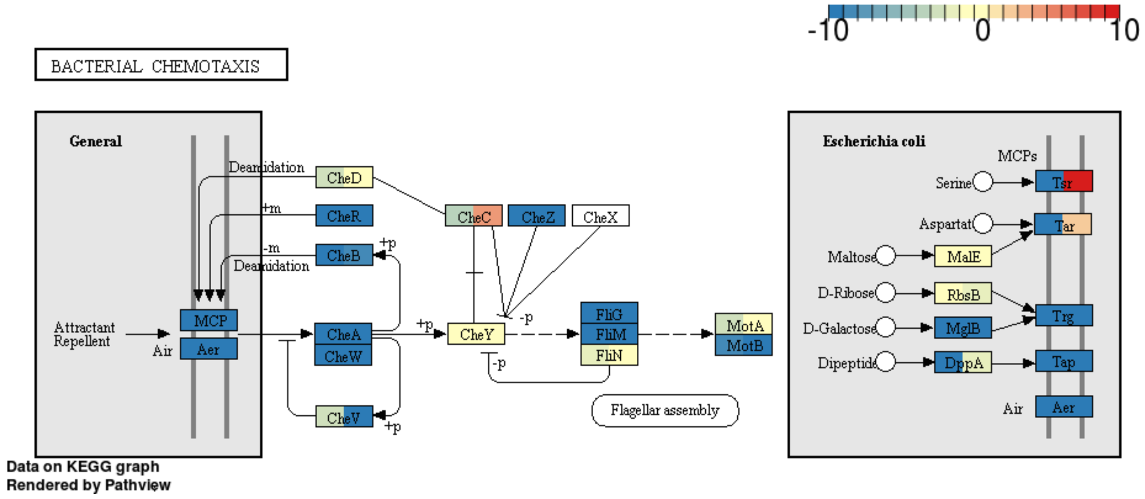

Supplementary Figure 9 | Underrepresentation of the bacterial chemotaxis pathway in caesarean-section-delivered neonates being born small for gestational age or not. Enzymes are depicted by boxes coloured according to the $\log_2$ fold changes (see colour key at top-right corner of the plot) obtained from the relative abundance of genes in gut metagenomes on days 3 and 5 in caesarean-section-delivered neonates (left part of the box) and additionally born small for gestational age (right part of the box), compared with vaginally delivered neonates. Log_2 fold changes were calculated with the R package 'DESeq2'[11] and the *pathview* R package[12] was used for plotting.

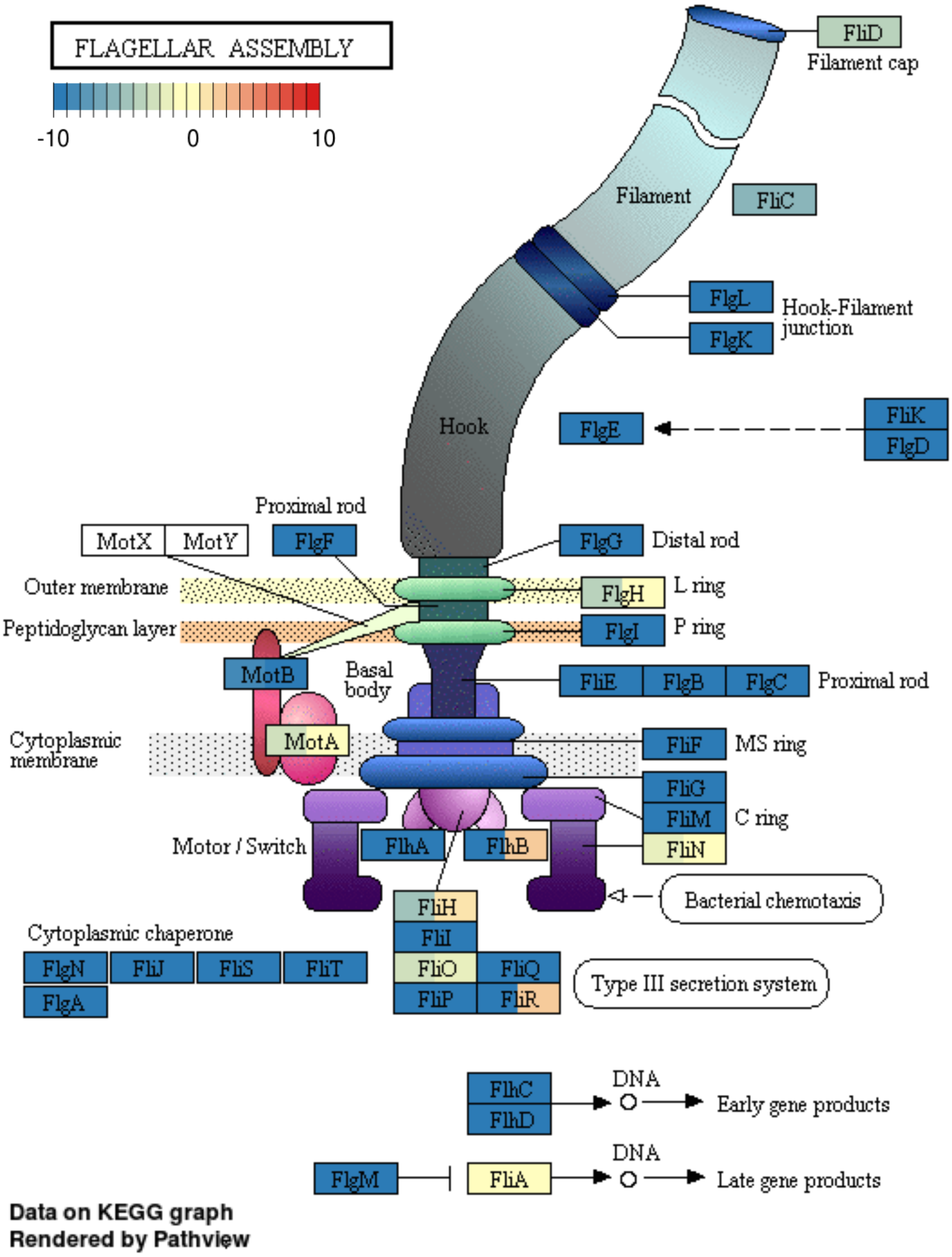

Supplementary Figure 10 | Underrepresentation of the flagellar assembly pathway in caesarean-section-delivered neonates being born small for gestational age or not. Enzymes are depicted by boxes coloured according to the $\log_2$ fold changes (see colour key at top-left corner of the plot) obtained from the relative abundance of genes in gut metagenomes on days 3 and 5 in caesarean-section-delivered neonates (left part of the box) and additionally born small for gestational age (right part of the box), compared with vaginally delivered neonates. Log_2 fold changes were calculated with the R package 'DESeq2'[11] and the *pathview* R package[12] was used for plotting.

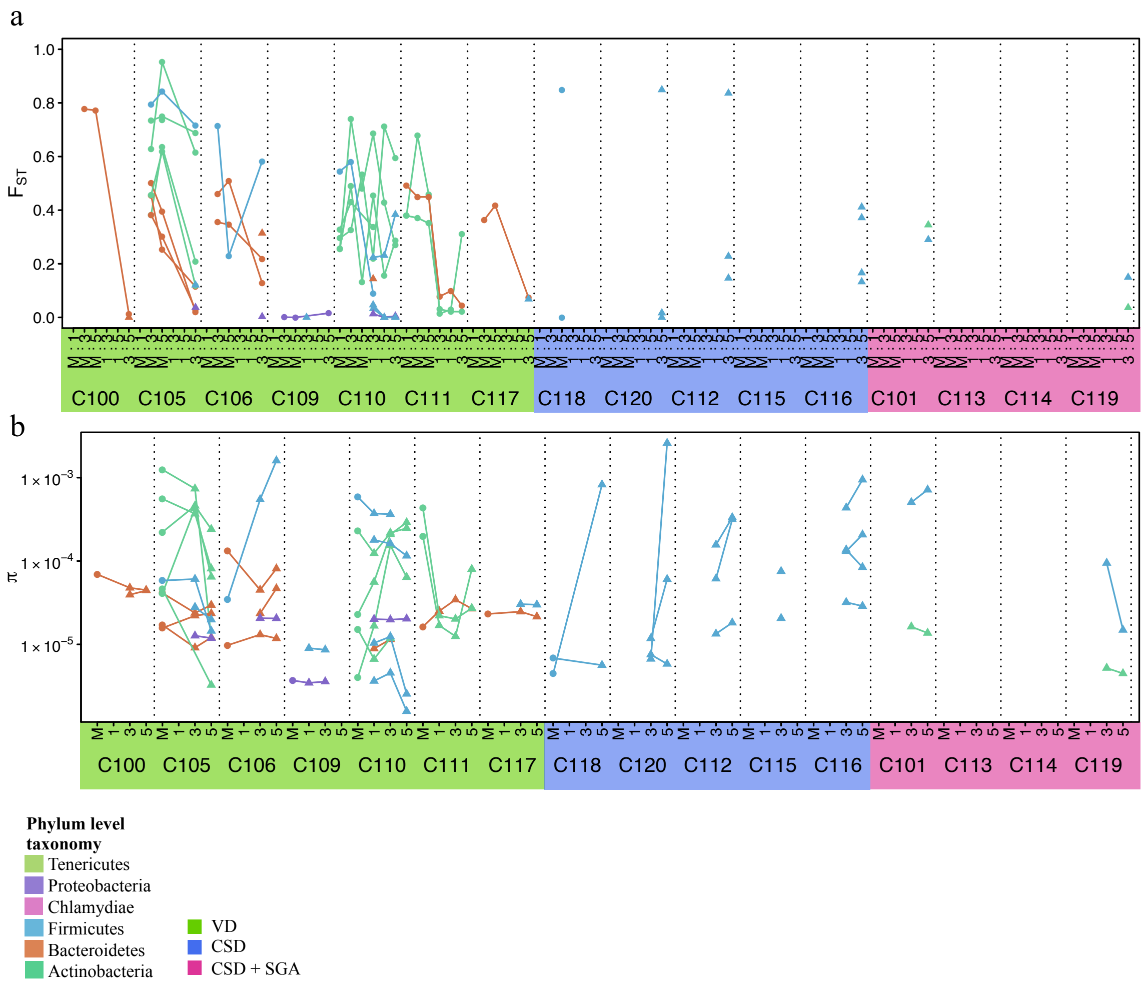

Supplementary Figure 11 | Genetic variation between microbial populations over time. a, Inter-population fixation index (F_{ST}) and **b**, intra-population diversity index (π) of populations, coloured by phylum and subdivided by mother–neonate pairs. Only microbial populations with genomes reconstructed to >65% completeness and with an existing link between samples are shown. Circles and triangles represent maternal and neonatal faecal samples, respectively. VD, vaginal delivery; CSD, caesarean-section delivery; SGA, small for gestational age; M, maternal faecal sample; 1/3/5, postnatal days of neonatal faecal sampling.

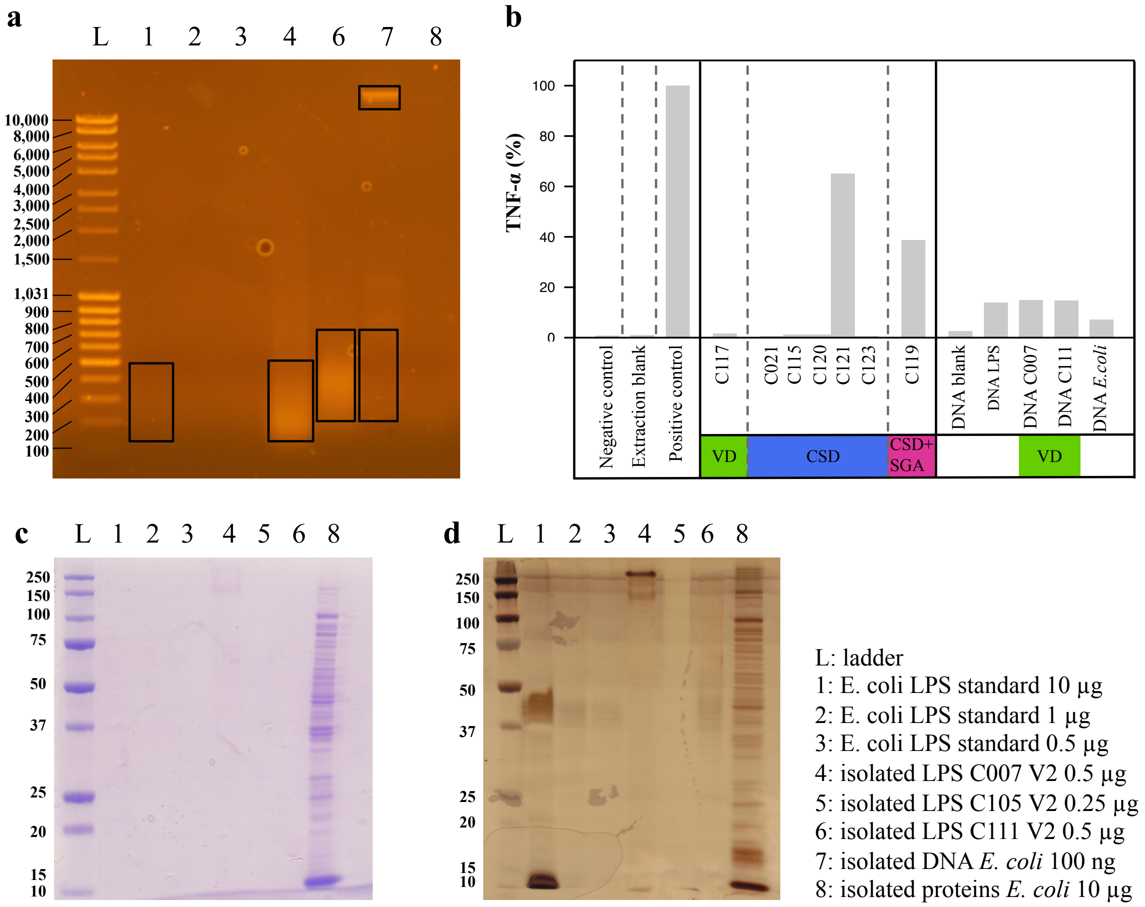

Supplementary Figure 12 | Purity assessment of isolated LPS fractions from neonatal stool. a, Agarose gel electrophoresis with Ethidium bromide staining to visualize the presence of DNA contamination in LPS fractions that were isolated from neonatal faecal samples. Agarose bands at the highlighted sizes were cut and DNA was isolated. **b,** Stimulation of human monocyte-derived dendritic cells (MoDCs) from adult donor 9 with extracted DNA samples obtained from **(a)**. Immune reaction was measured by levels of TNF-α in the supernatant. Untreated MoDCs were used as negative control, while MoDC stimulation with 15 endotoxin units (EU) of LPS isolated from an overnight culture of *Escherichia coli* strain K-12 (sub-strain MG1655) were used as positive control. **c,** Poly-acrylamide gel electrophoresis combined with Coomassie staining to visualize protein contamination. d, Poly-acrylamide gel electrophoresis combined with silver staining to visualize LPS presence.

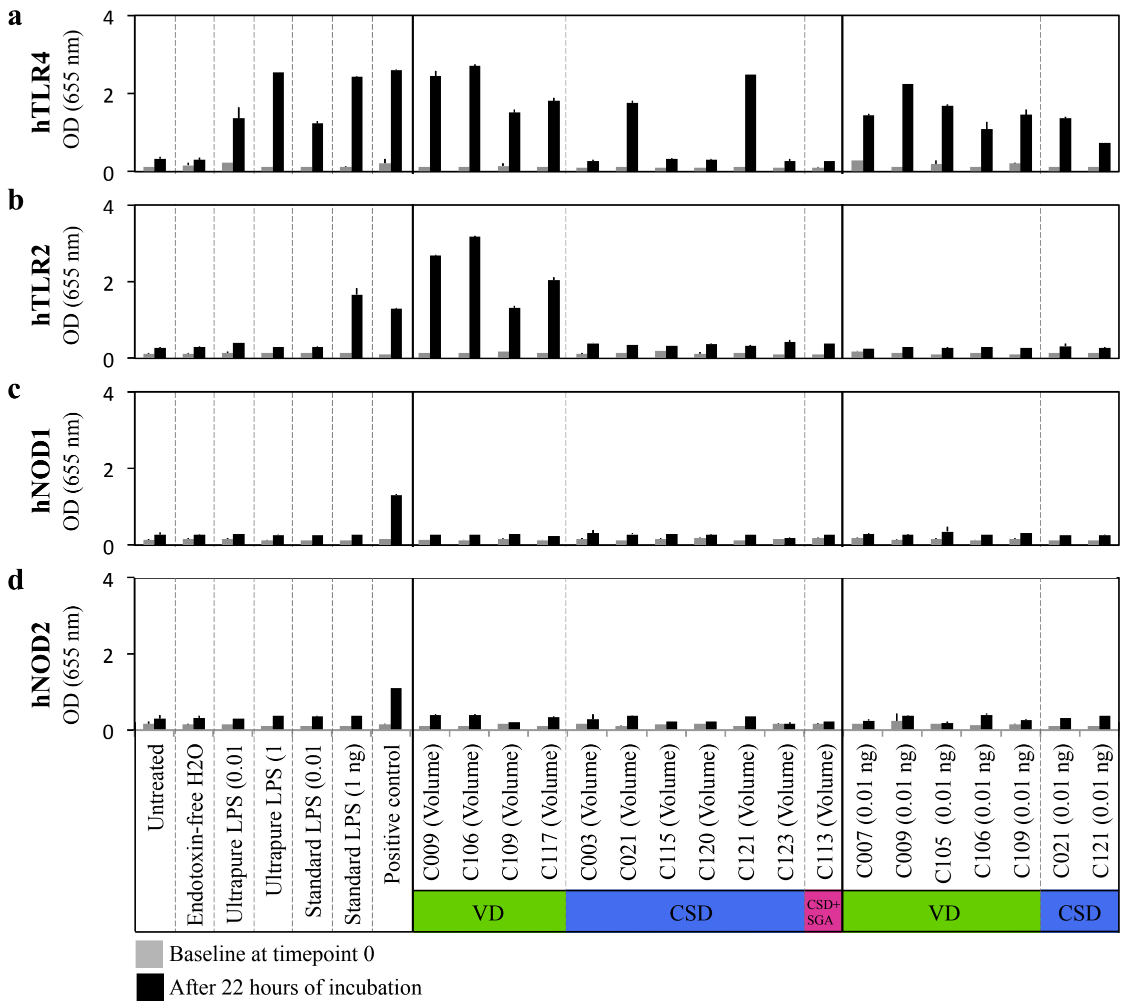

Supplementary Figure 13 | Immune stimulation assays of reporter cell lines after stimulation with isolated LPS from neonatal stool. Read-outs of HEK-Blue cell lines overexpressing respectively one of the membrane receptors hTLR4 **(a)**, hTLR2 **(b)**, NOD1 **(c)** or NOD2 **(d)** were stimulated with LPS fractions isolated from neonatal faecal samples. **a,** The hTLR4 receptor only recognizes LPS. Positive control: ultrapure LPS (5 µg). **b,** The hTLR2 receptor recognizes peptidoglycan, lipoteichoic acid and lipoprotein from gram-positive bacteria, lipoarabinomannan from mycobacteria, and zymosan from yeast cell wall. Positive control: Pam3CSK4 (1 µg). **c,** The NOD1 receptor binds to bacterial molecules containing the D-glutamyl-meso-diaminopimelic acid (iE-DAP) moiety. Positive control: TriDAP (10 µg). **d,** The NOD2 receptor recognizes bacterial molecules (peptidoglycans) and stimulates an immune reaction. Positive control: Murabutide (10 µg). OD: optical density. Technical duplicates were done per condition and error bars reflect the standard deviation between duplicates.

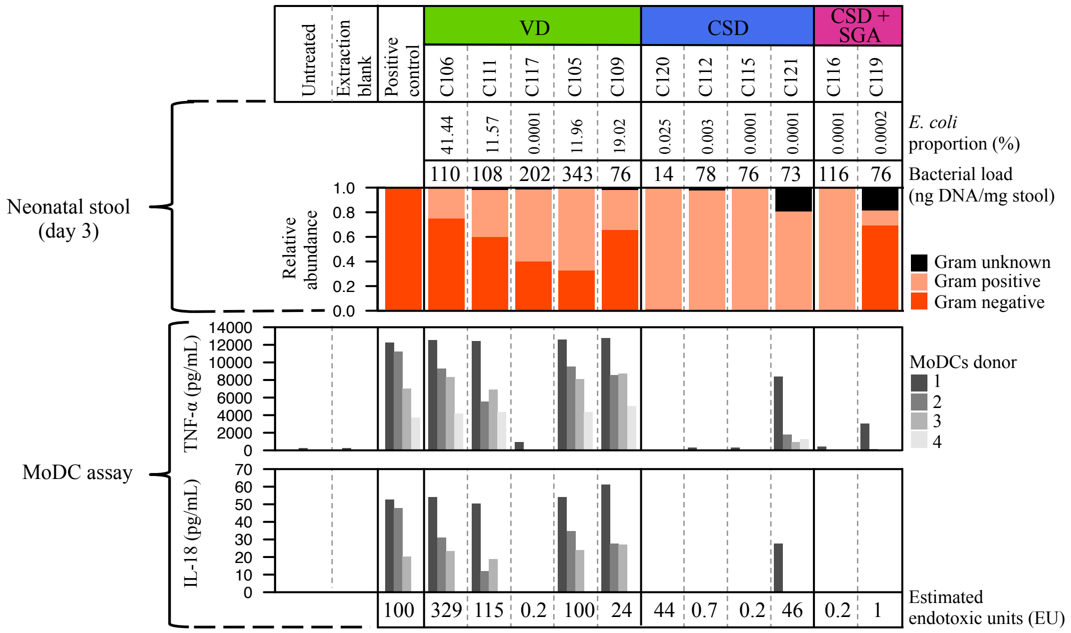

Supplementary Figure 14 | Cytokine profiles in monocyte-derived dendritic cells after stimulation with isolated LPS from neonatal stool and in neonatal plasma. Lipopolysaccharide (LPS) was isolated from faecal samples collected on day 3 postpartum from neonates in vaginal delivery (VD), caesarean-section delivery (CSD) and CSD with small for gestational age (SGA) status (CSD+SGA) groups, and incubated for 24 h with human monocyte-derived dendritic cells (MoDCs) isolated from four adult donors. The bacterial load was used for normalizing LPS concentration values. For neonate C120, only MoDCs from donor 4 were used, owing to the limited amount of isolated LPS. Neonates C115 and C116 are twins.

Supplementary References

1. Wampach, L. *et al.* Colonization and succession within the human gut microbiome by archaea, bacteria, and microeukaryotes during the first year of life. *Front. Microbiol.* **8,** (2017).
2. Laczny, C. C., Pinel, N., Vlassis, N. & Wilmes, P. Alignment-free visualization of metagenomic data by nonlinear dimension reduction. *Sci. Rep.* **4,** 4516 (2014).
3. Chu, D. M. *et al.* The early infant gut microbiome varies in association with a maternal high-fat diet. *Genome Med.* **8,** 77 (2016).
4. Perez-Muñoz, M. E., Arrieta, M.-C., Ramer-Tait, A. E. & Walter, J. A critical assessment of the "sterile womb" and "in utero colonization" hypotheses: implications for research on the pioneer infant microbiome. *Microbiome* **5,** (2017).
5. Salter, S. J. *et al.* Reagent and laboratory contamination can critically impact sequence-based microbiome analyses. *BMC Biol.* **12,** (2014).
6. Susilawati, T. N. *et al.* Deep sequencing approach for investigating infectious agents causing fever. *Eur. J. Clin. Microbiol. Infect. Dis.* **35,** 1137–49 (2016).
7. Morgan, X. C. *et al.* Dysfunction of the intestinal microbiome in inflammatory bowel disease and treatment. *Genome Biol.* **13,** R79 (2012).
8. Mandal, S. *et al.* Analysis of composition of microbiomes: a novel method for studying microbial composition. *Microb. Ecol. Health Dis.* **26,** 27663 (2015).
9. Vatanen, T. *et al.* Variation in Microbiome LPS Immunogenicity Contributes to Autoimmunity in Humans. *Cell* **165,** 842–853 (2016).
10. Sunagawa, S. *et al.* Metagenomic species profiling using universal phylogenetic marker genes. *Nat. Methods* **10,** 1196–1199 (2013).
11. Love, M. I., Huber, W. & Anders, S. Moderated estimation of fold change and dispersion for RNA-seq data with DESeq2. *Genome Biol.* **15,** 550 (2014).
12. Luo, W. & Brouwer, C. Pathview: an R/Bioconductor package for pathway-based data integration and visualization. *Bioinformatics* **29,** 1830–1 (2013).
