# Supplementary material for: Birth mode is associated with earliest strain-conferred gut microbiome functions and immunostimulatory potential
Source: Nat Commun. 2018 Nov 30;9:5091. doi: 10.1038/s41467-018-07631-x (PMC6269548; doi:10.1038/s41467-018-07631-x)
Supplement: Supplementary file 3 — Description of Additional Supplementary Files [file 41467_2018_7631_MOESM3_ESM.docx]

**Description of Additional Supplementary Files**

File Name: Supplementary Data 1

Description: Sample- and datatypes. List of collected metadata and performed

analyses, including different types of sequencing, lipopolysaccharide (LPS) characterization

and blood plasma analyses, for all samples.

File Name: Supplementary Data 2

Description: Stats on metagenomics data. Detailed information on the steps of

sequence filtering according to quality, removal of human reads and removal of putative

contaminant sequencing reads.

File Name: Supplementary Data 3

Description: mOTU - Weighted read counts. Numbers of reads mapping to

metagenomic operational taxonomic units (mOTUs) per study sample.

File Name: Supplementary Data 4

Description: KEGG orthology - Read counts. Numbers of metagenomic reads

mapping to assembled genes representing KEGG orthologous groups per study sample.

File Name: Supplementary Data 5

Description: Genome reconstructions. Statistics on reconstructed genomes and

represented functional pathways according to delivery mode.

File Name: Supplementary Data 6

Description: 16SrRNA - Read counts. Numbers of reads mapping to 16S rRNA

gene amplicon-based operational taxonomic units (OTUs) per study sample.

File Name: File Name: Supplementary Data 7

Description: mOTU statistics. Statistics on mOTUs according to delivery mode

and small for gestational age status using multiple Wilcoxon rank sum tests.

File Name: Supplementary Data 8

Description: 16S rRNA gene sequencing statistics. Statistics on OTUs

according to delivery mode and small for gestational age status using multiple Wilcoxon rank

sum tests.

File Name: Supplementary Data 9

Description: MaAsLin summary table. Multivariate analyses for 16S rRNA

gene amplicon and metagenomic sequencing datasets taking into account multiple collected

variables per study participant.

File Name: Supplementary Data 10

Description: KOs DESeq2. Significantly differential functional categories

according to delivery mode as detected by DESeq2.

File Name: Supplementary Data 11

Description: PanFP predictions. 16S rRNA gene-based predicted functional

categories per study sample.

8

File Name: Supplementary Data 12

Description: LPS isolation. LPS isolation and quantification from neonatal

stool samples collected at day 3 after delivery.

File Name: Supplementary Data 13

Description: LPS stimulation assays TNFa. Measured levels of LPS-triggered

TNFa produced by monocyte-derived dendritic cells.

File Name: Supplementary Data 14

Description: qPCR primer sequences. Primer sequences for quantitative realtime

PCR and bacterial yield determination.

File Name: Supplementary Data 15

Description: Load of bacteria and LPS. Bacterial load and LPS levels per

sample.

File Name: Supplementary Data 16

Description: LPS-triggered cytokines. Levels of LPS-triggered cytokines

produced by monocyte-derived dendritic cells, measured by Magpix.

File Name: Supplementary Data 17

Description: Plasma cytokines. Measured levels of cytokines in neonatal blood

plasma.
