# Supplementary material for: Birth mode is associated with earliest strain-conferred gut microbiome functions and immunostimulatory potential
Source: Nat Commun. 2018 Nov 30;9:5091. doi: 10.1038/s41467-018-07631-x (PMC6269548; doi:10.1038/s41467-018-07631-x)
Supplement: Supplementary file 21 — Reporting Summary [file 41467_2018_7631_MOESM21_ESM.pdf]

nature research

Corresponding author(s): Paul Wilmes

☐ Initial submission ☐ Revised version ☒ Final submission

Life Sciences Reporting Summary

Nature Research wishes to improve the reproducibility of the work that we publish. This form is intended for publication with all accepted life science papers and provides structure for consistency and transparency in reporting. Every life science submission will use this form; some list items might not apply to an individual manuscript, but all fields must be completed for clarity.

For further information on the points included in this form, see Reporting Life Sciences Research. For further information on Nature Research policies, including our data availability policy, see Authors & Referees and the Editorial Policy Checklist.

Experimental design

1. Sample size

Describe how sample size was determined.

In the framework of this pilot study, we analysed data from 33 neonates, including 16 newly enrolled mother-neonate pairs. Per study group, at least 4 mother-neonate pairs were included (vaginal delivery, caesarean section delivery and caesarean section delivery + born small for gestational age).

2. Data exclusions

Describe any data exclusions.

Exclusion criteria for enrollment of mother–neonate pairs included the administration of antibiotics to neonates immediately postpartum, birth prior to 34 weeks of gestation, and maternal gestational diabetes.

3. Replication

Describe whether the experimental findings were reliably reproduced.

not applicable

4. Randomization

Describe how samples/organisms/participants were allocated into experimental groups.

Samples were allocated per study group dependent on the delivery mode of the neonate and their small for gestational age status. Additional clinical data were recorded per mother-neonate pair.

5. Blinding

Describe whether the investigators were blinded to group allocation during data collection and/or analysis.

Investigators were not blinded to group allocation during data collection and analysis.

Note: all studies involving animals and/or human research participants must disclose whether blinding and randomization were used.

6. Statistical parameters

For all figures and tables that use statistical methods, confirm that the following items are present in relevant figure legends (or in the Methods section if additional space is needed).

n/a	Confirmed	
☐	☒	The exact sample size (n) for each experimental group/condition, given as a discrete number and unit of measurement (animals, litters, cultures, etc.)
☐	☒	A description of how samples were collected, noting whether measurements were taken from distinct samples or whether the same sample was measured repeatedly
☐	☒	A statement indicating how many times each experiment was replicated
☐	☒	The statistical test(s) used and whether they are one- or two-sided (note: only common tests should be described solely by name; more complex techniques should be described in the Methods section)
☐	☒	A description of any assumptions or corrections, such as an adjustment for multiple comparisons
☐	☒	The test results (e.g. P values) given as exact values whenever possible and with confidence intervals noted
☐	☒	A clear description of statistics including central tendency (e.g. median, mean) and variation (e.g. standard deviation, interquartile range)
☐	☒	Clearly defined error bars

See the web collection on statistics for biologists for further resources and guidance.

Software

Policy information about availability of computer code

7. Software

Describe the software used to analyze the data in this study.	All custom scripts written for this study and used softwares are publicly available online at https://git-r3lab.uni.lu/Cosmic/Earliest .

For manuscripts utilizing custom algorithms or software that are central to the paper but not yet described in the published literature, software must be made available to editors and reviewers upon request. We strongly encourage code deposition in a community repository (e.g. GitHub). *Nature Methods* guidance for providing algorithms and software for publication provides further information on this topic.

Materials and reagents

Policy information about availability of materials

8. Materials availability

Indicate whether there are restrictions on availability of unique materials or if these materials are only available for distribution by a for-profit company.	No unique materials were used.

9. Antibodies

Describe the antibodies used and how they were validated for use in the system under study (i.e. assay and species).	Some antibodies used in the present study were purchased from R&D Systems within the following kits:<br>1) LXSAHM-18: Luminex Screening Human Magnetic Assay<br>CXCL8/IL-8 (BR18), Galectin-1 (BR51), Granzyme B (BR57), IFN-beta (BR21), IFN-gamma (BR29), IL-1 beta (BR28) IL-10 (BR22) IL-12/23 p40 (BR67) IL-13 (BR47) IL-15 (BR63) IL-18 (BR78) IL-2 (BR43) IL-21 (BR65) IL-27 (BR25) IL-4 (BR39) IL-5 (BR53) IL-6 (BR13) TNF-alpha (BR12)<br><br>2) LXSAHM-7:Luminex Screening Human Magnetic Assay<br>CXCL8/IL-8 (BR18); IL-1 beta (BR28); IL-10 (BR22); IL-12 p70 (BR56); IL-18 (BR78); IL-6 (BR13); TNF-alpha (BR12)<br><br>Other antibodies used in the present study were purchased from Life Technologies within the following kit: human TNF alpha uncoated ELISA

10. Eukaryotic cell lines

a. State the source of each eukaryotic cell line used.

- The human epithelial colorectal cell line Caco-2 cells was purchased from DSMZ (ACC169) and these cells were grown in Dulbecco's Modified Eagle's Medium containing 20% v/v foetal bovine serum.
- HEK-Blue™ hTLR4 was purchased from Invivogen. HEK-Blue™-hTLR4 cells were obtained by co-transfection of the human TLR4, MD-2 and CD14 co-receptor genes, and an inducible SEAP (secreted embryonic alkaline phosphatase) reporter gene into HEK293 cells. The SEAP reporter gene is placed under the control of an IL-12 p40 minimal promoter fused to five NF-κB and AP-1-binding sites. Stimulation with a TLR4 ligand activates NF-κB and AP-1 which induce the production of SEAP. Levels of SEAP can be easily determined with HEK-Blue™ Detection, a cell culture medium that allows for real-time detection of SEAP.
The parental cell line of HEK-Blue™ hTLR4 cells is HEK-Blue™ Null2 cells.
- HEK-Blue™-hTLR2 cells were obtained by co-transfection of the human TLR2 and SEAP genes into HEK293 cells. The SEAP reporter gene is placed under the control of the IFN-β minimal promoter fused to five NF-κB and AP-1-binding sites. Additionally, the CD14 co-receptor gene was transfected into these cells to enhance the TLR2 response. Stimulation with a TLR2 ligand activates NF-κB and AP-1 which induce the production of SEAP.
The parental cell line of HEK-Blue™ hTLR2 cells is HEK-Blue™ Null1 cells.
- HEK-Blue™-hNOD1 cells were obtained by co-transfection of the human NOD1 gene and an optimized SEAP reporter gene into HEK293 cells. The SEAP reporter gene is placed under the control of the IFN-β minimal promoter fused to five NF-κB and AP-1 binding sites. Stimulation with a NOD1 ligand activates NF-κB and AP-1 which induce the production of SEAP.
The parental cell line of HEK-Blue™ hNOD1 cells is HEK-Blue™ Null1 cells.
- HEK-Blue™-hNOD2 cells were obtained by co-transfection of the human NOD2 gene and an optimized SEAP reporter gene into HEK293 cells. The SEAP reporter gene is placed under the control of the IL-12 p40 minimal promoter fused to five NF-κB and AP-1 binding sites. Stimulation with a NOD2 ligand activates NF-κB and AP-1 which induce the production of SEAP. The parental cell line of HEK-Blue™ hNOD2 cells is HEK-Blue™ Null2 cells.

b. Describe the method of cell line authentication used.

- DSMZ certifies that fluorescent nonaplex PCR of short tandem repeat markers revealed a unique STR DNA profile.
- HEK-Blue hTLR4: Expression of human TLR4 and MD2/CD14 genes has been confirmed by RT-PCR. The cell surface expression of human TLR4 in this cell line has been validated using fluorescence-activated cell sorting (FACS). The stability of this cell line for 20 passages following thawing has been verified.
- HEK-Blue hTLR2: The expression of human TLR2 has been confirmed by FACS. The expression of the human TLR2 and CD14 genes has been confirmed by RT-PCR. The stability of this cell line for 20 passages following thawing has been verified.
- HEK-Blue Nod1: the expression of human NOD1 has been confirmed by RT-PCR. The stability of this cell line for 20 passages following thawing has been verified.
- HEK-Blue Nod2: the expression of human NOD2 has been confirmed by RT-PCR. The stability of this cell line for 20 passages following thawing has been verified.

c. Report whether the cell lines were tested for mycoplasma contamination.

-Caco-2 cells have been tested for mycoplasma contamination by DSMZ and ourselves and no contamination has been observed.
- All HekBlue cell lines are guaranteed mycoplasma-free by Invivogen.

d. If any of the cell lines used are listed in the database of commonly misidentified cell lines maintained by ICLAC, provide a scientific rationale for their use.

not applicable

Animals and human research participants

Policy information about studies involving animals; when reporting animal research, follow the ARRIVE guidelines

11. Description of research animals

Provide details on animals and/or animal-derived materials used in the study.

No animals were used.

June 2017

Policy information about studies involving human research participants

12. Description of human research participants

Describe the covariate-relevant population characteristics of the human research participants.	Please refer to 'Methods' sections ' Ethics' and 'Clinical metadata' in the main manuscript.
